# Biomechanical Design and Prototyping of a Powered Ankle-Foot Prosthesis

**DOI:** 10.3390/ma13245806

**Published:** 2020-12-19

**Authors:** Stefano Alleva, Michele Gabrio Antonelli, Pierluigi Beomonte Zobel, Francesco Durante

**Affiliations:** Dipartimento di Ingegneria Industriale e dell’Informazione e di Economia—DIIIE, Università dell’Aquila, Monteluco di Roio, 67100 L’Aquila, Italy; stefal639@gmail.com (S.A.); gabrio.antonelli@univaq.it (M.G.A.); francesco.durante@univaq.it (F.D.)

**Keywords:** biomechanical design, ankle-foot prosthesis, experimental validation

## Abstract

Powered ankle-foot prostheses for walking often have limitations in the range of motion and in push-off power, if compared to a lower limb of a healthy person. A new design of a powered ankle-foot prosthesis is proposed to obtain a wide range of motion and an adequate power for a push-off step. The design methodology for this prosthesis has three points. In the first one, a dimensionless kinematic model of the lower limb in the sagittal plane is built, through an experimental campaign with healthy subjects, to calculate the angles of lower limb during the gait. In the second point a multibody inverse dynamic model of the lower limb is constructed to calculate the foot-ground contact force, its point of application and the ankle torque too, entering as input data the calculated angles of the lower limb in the previous point. The third point requires, as input of the inverse dynamic model, the first dimensioning data of the ankle-foot prosthesis to obtain the load acting on the components of the prosthesis and the angle torque of the actuator during the gait cycle. Finally, an iteration cycle begins with the inverse dynamic model modifying the ankle torque and angle until these quantities during the gait are as close as possible to the physiological quantities. After the mechanical design and the construction of the prototype of the prosthesis, an experimental methodology was used for preliminary validation of the design. The preliminary tests in the laboratory on the prototype alone show that the range of motion of the ankle angle during the gait is close to a healthy person’s: 27.6° vs. 29°. The pushing force of the distal area of the prototype is 1.000 N, instead of 1.600 N, because a budget reduction forced us to choose components for the prototype with lower performance.

## 1. Introduction

Prostheses are designed to allow amputees to return to a satisfying social and working life. Many prostheses, particularly those for individuals with transtibial amputations, do not correctly reproduce the physiological function of the joints and lead to a gait with functional limitations. People with these amputations may have decreased walking speed and increased asymmetries between the lower limbs in step length, swing time, when the foot is in contact with the ground and there is a reciprocal exchange of energy, and stance time, when the foot is not in contact with the ground. To optimally reproduce the function of the ankle joint during walking, prostheses for people with transtibial amputations must well-approximate the range of motion of the ankle angle, the curve of torque, and power at the ankle during gait.

Until the early 2000s, commercially available ankle-foot prostheses were completely passive during the gait, and, consequently, the mechanical properties in the stance-phase did not adapt to the walking speed and the type of terrain. In the following years, researchers and commercial companies proposed the energy storing returning foot (ESR foot), an ankle-foot prosthesis with integrated systems for storing and releasing energy during the stride [1,2]. The ESR foot is able to store energy during the stance phase and release it later during the push-off step [3]. The push-off step [4] is the period of positive push-off limb power near the terminal stance-phase and immediately prior to the foot-lift, when the weight-accepting foot is being lowered to the ground.

**Figure 1:**
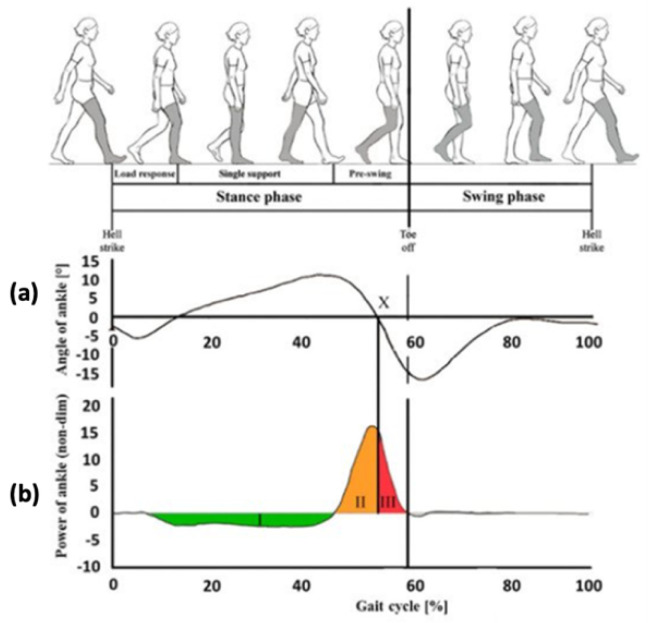
(**a**) Diagram of the ankle angle in the gait of a healthy individual and (**b**), corresponding diagram of the power of the ankle, rendered dimensionless. I is the area with negative power, starting from the heel strike; II is an amount of the area with positive power corresponding to the thrust phase; III is the remaining amount of the area with positive power, corresponding at the final stage of the stance phase in a healthy individual; X is the point in which the foot loses contact with the ground, in the case of a user with an ESR foot.

Figure 1 shows, with regard to phases and sub-phases of physiological walking, the curve of the ankle angle, in (a), and the curve of the power received and supplied to the physiological ankle, in (b). In Figure 1a, at point X, the foot loses contact with the ground and this makes it impossible to exchange any force. In Figure 1b three distinct areas are visible. Area I, which starts from the heel strike, is an area with negative power, and represents the amount of energy stored within the muscle and tendons in the physiological ankle and within the elastic element in the prosthesis. Area II is a positive power area and represents the thrust phase, when the previously stored energy is released. Areas I and II have approximately the same extension. Finally, there is area III, in continuity with II at coordinate X (areas II plus III represent the push-off step), which is not present in the case of a user with an ESR foot, because at this coordinate there is a loss of contact with the ground. Thus, a person with an ESR foot shows a trend of the ankle angle that is different from the physiological one.

It is important to point out that the human ankle is known to vary the impedance in the gait-cycle, passing from walking on level ground to climbing and then descending, possibly through a staircase [5,6,7,8]. Furthermore, numerous studies have made it clear that one of the main functions of the human ankle is to provide adequate energy for the forward propulsion of the body, during the push-off step. Therefore, only a powered ankle-foot prosthesis could allow for the accumulation and return of more energy than an ESR foot, allowing the amputee to have a walk closer to the physiological one.

More recently, prototypes of powered ankle-foot prostheses have been developed with a combination of energy recovery and motorized systems for the development of the torque to be applied to the ankle [9,10,11]. Typically, electric power is widely adopted for these prostheses, but non-traditional pneumatic actuators have also been proposed [12,13]. A recent review of active lower limb prosthetics [14] found nine below-knee powered prostheses, realized as physical prototypes, that add to the three commercial ones (SpringActive, BionX, and Freedom Innovations). Many actuation systems make use of compliant elements, such as springs, which can also implement variable elastic properties to obtain a variable impedance actuation system. Furthermore, some of the powered ankle-foot prostheses do not have the same range of motion as the ankle angle in a healthy individual [6,8,9] and in some cases the push-off power is less than in the physiological one [6,8,9,11].

The aim of this paper is to present the biomechanical design and the construction of a first prototype of an innovative powered ankle-foot prosthesis with energy storage inside. A preliminary experimental validation, after the testbed design, is also described in the paper. The goal of the authors with the new design is to obtain a powered ankle-foot prosthesis with a wide range of motion and adequate power for a push-off step in order to perform a level-ground gait close to the physiologically correct one. Moreover, the methodology followed for the design of this kind of prosthesis is applied [15] and it may also be useful for other researchers in this field. The prosthesis consists of five-link device integrated with two keys elements: a shock-absorber and a linear actuator-transmissions group. During the stance phase, the simultaneous operation of the shock-absorber and of the actuator allows for the recovery and transfer of energy for the push-off step; during the swing phase, the ankle angle is adjusted by the action of the actuator only. The proposed ankle-foot prosthesis has the ankle angle continuously adjusted in both phases of the gait and the simultaneous operation of the two elements, the actuator-transmissions group and shock-absorber, allows the generation of the necessary ankle torque in the propulsion phase of the gait.

## 2. Methods, Design, and Prototyping

### 2.1. Design Methodology for the Ankle-Foot Prosthesis

The methodology used for the design of the ankle-foot prosthesis has three points. Point 1 requires the construction of a dimensionless model of the lower limb that serves to represent the physiological gait in the sagittal plane. This model is built through an experimental campaign that involved a sample of healthy subjects. Subjects with no history of musculoskeletal or neurologic impairment were recruited and enrolled among researchers and PhD students (Table 1). An informed written consent was obtained from each participant. Subjects must walk on a treadmill. Four markers are placed on the subjects (hip, knee, ankle, and front of the shoe). A stereophotogrammetric analysis was used in order to detect the trajectories of these markers and, with post-processing, the values of the 3 characteristic angles in the sagittal plane: dorsiflexion of the foot, flexion of the knee, and flexion of the hip.

**Figure 2:**
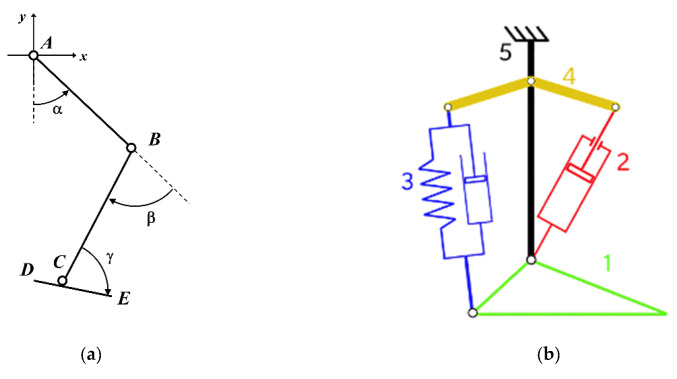
(**a**) The lower limb with the 3 angles and the mobile reference frame and, (**b**), the sketch of the ankle-foot prosthesis designed. About the components of the prosthesis: 1 is the foot; 2 is the linear actuator; 3 is the shock-absorber; 4 is the rocker arm; 5 is the prosthetic tibia.

The calculated angles are post-processed to refer to a mobile reference frame, centered in the hip joint, for α, while β and γ are the angles, respectively, between the femur and tibia and between the tibia and foot (Figure 2a). A dimensionless parametric model of the lower limb is then created, by Mathworks^®^ MATLAB environment, using the calculated values of these angles. The model thus constructed has the average size of the sample of the subjects whose gait has been the subject of experimental analysis. The model parameters are the size of the femur (AB), tibia (BC), and foot (DE). For the femur and tibia the dimensions are represented by the distance between the hinges, while for the foot its length, and the center of the joint is placed at 1/3 of the total length: CE = 2/3 DE. The dimensionless model thus constructed is used to simulate the gait in a plane, starting from the contact position of the heel with the ground and ending when the same position is reached, that is, after the same leg has completed the step.

Point 2 of this methodology requires the construction of a multibody inverse dynamic model of the lower limb based on the rigid body model. The input data are the angles of the hip, knee, and ankle joints (calculated in point 1), the lengths of the femur, tibia, and foot, and the masses, considering an 80-kg subject. The output of this model is the resulting foot/ground contact force, its point of application, and finally the ankle torque in the gait cycle. This result depends on the mass and the anthropometric dimensions of the subject; the gait analysis software C-Motion Visual3D Professional, during a physiological correct gait simulation, has been used to define the model.

After this point, the architecture of the powered ankle-foot prosthesis was defined and the new design is shown in Figure 2b as a sketch. It is made of a foot (1) and a tibia (5), joined together by a rocker arm (4) used as fastening element for the actuator (2) and the shock-absorber (3). The architecture of the prosthesis was defined to obtain a gait behavior as close as possible to the physiological one. The combined action of the shock absorber and actuator allows for a wide range of motion of the ankle and an adequate ankle torque for push-off step.

Finally, point 3 requires the modification of the multibody inverse dynamic model of the lower limb by inserting the ankle-foot prosthesis and neglecting inertia actions, due to their modest influence in a normal and regular gait. By using this model, it is possible to calculate the following, in a physiological gait cycle on flat ground at a walking speed of about 1.5 m/s:

1. The loads acting on the individual components of the prosthesis.
2. The ankle torque of the actuator in the gait cycle. The torque law and the displacement law of the ankle, obtained from the previous phases for a physiological step, are inserted in the prosthesis model of the lower limb. Once the step obtained with this model has been calculated, the torque law and the ankle displacement law are iteratively modified until the gait is as close as possible to the physiological one.

The proposed ankle-foot prosthesis has the following characteristics in the execution of the step. Stance phase: the position of the ankle is regulated by the physiological dynamics of the step as in a healthy individual. The torque of the ankle is generated by the elastic force of the spring and therefore depends on its length. Once the ankle angle value and the required torque have been defined, the actuator modifies the length of the spring by adjusting the torque at the ankle. *Swing phase*: the ankle angle is controlled by the length of the actuator throughout the swing phase, so as to prevent the person from stumbling.

### 2.2. Ankle-Foot Prosthesis Design and Prototyping

**Figure 3:**
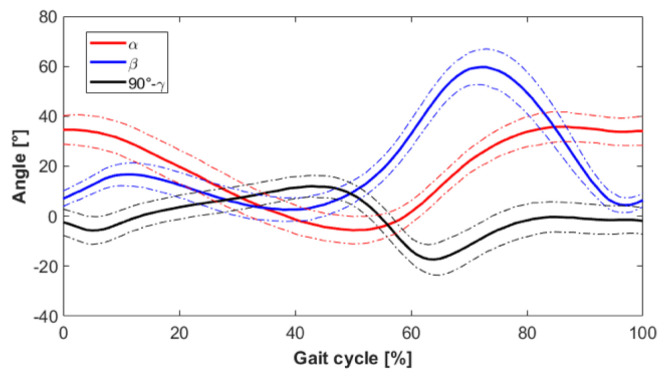
The diagram of the 3 angles of the lower limb during the gait (solid lines show the average values of the angles; dashed lines the standard deviation range): hip angle α, knee angle β, and ankle angle γ.

Following the methodology previously illustrated in point 1, the results of Figure 3 have been obtained in terms of the pattern of the angles characteristic of the lower limb during the walk for the recruited subjects (Table 1).

**Figure 4:**
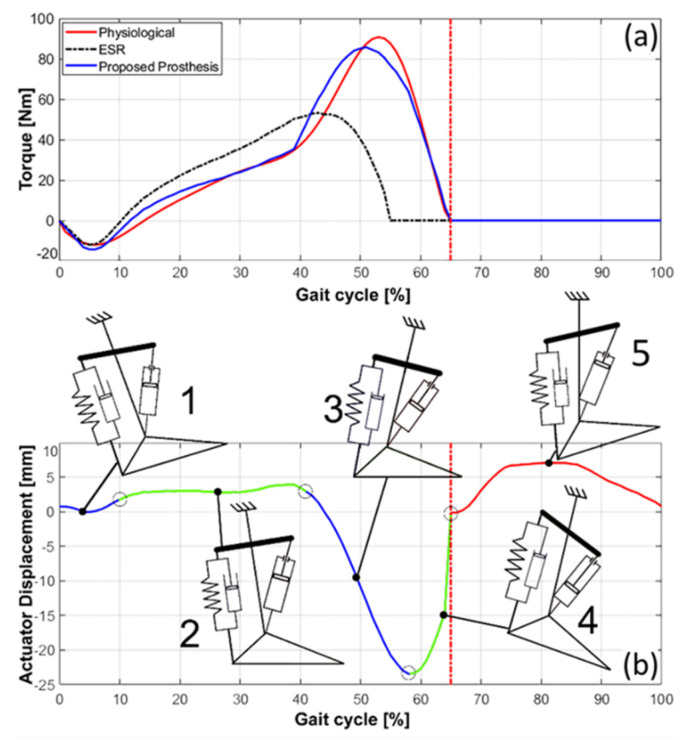
(**a**) The diagram of the ankle torque for 3 different gait conditions: the physiological gait, the one with the ESR foot and the one with the modeling based on the proposed prosthesis and, (**b**), the diagram of the actuator displacement and configuration of the ankle-foot prosthesis: 1 is the heel strike sub-phase; 2 is the foot-flat sub-phase; 3 is the start of the pre-swing phase; 4 is the ending of the pre-swing sub-phase when the foot detaches from the ground; 5 is the swing phase.

Then using these characteristic angles, through the inverse dynamic multybody model, point 2, the diagram of the ankle torque during the walk was determined: Figure 4a shows the diagram of the ankle torque obtained in comparison with the torque calculated with the same model for the proposed ankle-foot prosthesis and the torque delivered by an ESR foot. Figure 4b shows the length of the actuator in the different points of the step and the corresponding configuration of the prosthesis.

It is interesting to comment on some evidence that emerges from Figure 4b. Moving from position 1, heel strike, to position 2, it is observed that the torque at the ankle first reduces and then begins a sustained growth, while the length of the actuator grows slightly. Once past position 2, the torque maintains the same growth and then accelerates it to position 3, the beginning of the pre-swing phase, where the torque reaches its maximum value. The length of the actuator changes in the second phase of torque growth, with a specular decrease in length, as expected, since the actuator is primarily responsible for the length of the spring that applies the torque to the ankle. After position 3, the torque decreases as does the length of the actuator due to the increase in the ankle angle. The length of the actuator then reaches its minimum value and then increases again while the torque of the ankle continues to decrease until it reaches a value close to zero at position 4 of the prosthesis, in which the foot detaches from the ground.

**Figure 5:**
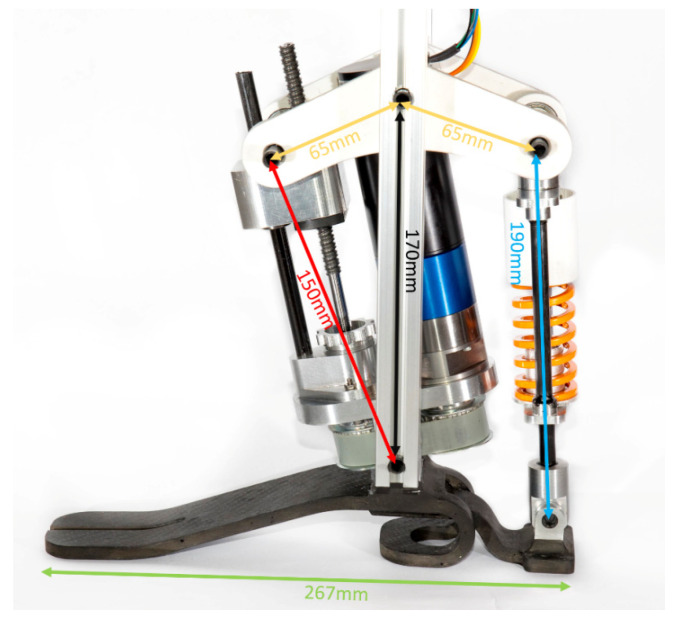
An image of the prototype of the ankle-foot prosthesis with the main dimensions (the actuator and shock-absorber dimensions refer to the condition at γ = 90°).

The prototype of the ankle-foot prosthesis has been designed for an 80-kg-mass subject, corresponding to the 75th percentile of the Italian adult population, and it is designed to carry out the preliminary validation tests in the laboratory without a patient. The anthropometric parameters have been associated according to ISO 7250-1: 2017 [16] and the load parameters have been referred to the objective physical activity identified by the 4th K-level of the Medicare Functional Classification Level (MFCL) [17]. The main dimensions of the ankle-foot prosthesis prototype are shown in Figure 5, where the prototype is shown. For manufacturing the prototype, the aluminum alloy ALU 6082-T6 has been used for a rocker arm, the main parts of the shock-absorber and the connecting parts of the electrical motor and transmissions. A pair of carbon fiber skin-core structures, with connecting parts in the same aluminum alloy drowned in carbon fiber, has been used for the foot.

The multibody dynamic model has been used to calculate the maximum loads on the prosthesis by entering the forces that the ground transmits to the foot in a gait cycle, Table 2 and Table 3. These load conditions have been used for the dimensioning of the components of the prototype

#### 2.2.1. Foot Design and Prototyping

**Figure 6:**
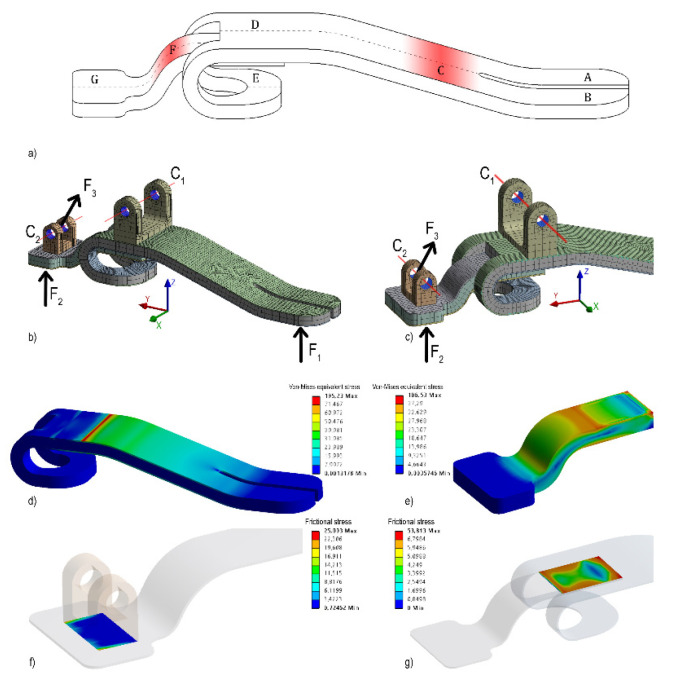
(**a**) Geometry of the prosthetic foot: A and B are the left and right distal areas, rispectively; C is the metatarsal area; D is the tarsal area; E is the tarsal support area; G is the calcaneal area; (**b**,**c**) finite element model of the foot: mesh and applied loads (F1 = 2.000 N, F2 = 2.000 N, F3_z_ = 1.835 N, F3_y_ = −490 N); equivalent stresses: (**d**) for a critical distal load, (**e**) for a critical calcaneal load; frictional stresses: (**f**) on the glued calcaneal area; (**g**) on the glued area between the two skin-core structures.

After the functional design of the ankle-foot prosthesis, a detailed design of the foot was carried out before manufacturing the prototype in a carbon fiber skin-core structure. The prosthetic foot needs to have characteristics similar to a physiological foot: high flexural stiffness in the tarsal area, to reduce deformations and to give stability to the user, compliance in the metatarsal area, to improve the grip on slippery and/or uneven grounds, and compliance in the heel area, to limit possible impact stresses. The main dimensions of the proposed prosthetic foot, according to [16], are the length of the foot and the height of the malleolus fixed to 267 and 60 mm, respectively. The longitudinal position of the latter has been imposed at 1/3 of the total length of the foot, starting from the heel side. The geometry of the foot, made of a pair of carbon fiber skin-core structures, is shown in Figure 6a, where these parts can be located: (A) left distal area, (B) right distal area, (C) metatarsal area, (D) flat tarsal area and (E) tarsal support area, which constitute the first skin-core structure; (F) curvilinear tarsal area and (G) calcaneal area, which constitute the second skin-core structure. The finite element modeling, Figure 6b–g, of the carbon fiber skins and the foam core by the Ansys Composite PrepPost (ACP) module of the Ansys 19.2 code has been used for the dimensioning.

The stress analysis of the skin-core structures has been carried out for two different critical load conditions (Figure 6b,c): a lumped load on the distal area (F1) and a lumped load on the calcaneal area (F2). The F1 and F2 values have been set from the impact equivalent condition described in the MFCL [17]. For the first load condition, hinge-type constraints have been placed on hinges C1 and C2; for the second load condition, a fixed constraint has been applied at C1. In the latter load condition, the bonding between the two structures has been also checked. To check the bonding in the heel area, the lumped load F3 and a fixed constraint at C1 have been applied. The bonding in the flat tarsal area has not been checked due to the action of the compression loads. The Tsai-Wu criterion has been adopted for the equivalent stress analysis in the skins. The criterion of τ_lim_ = τ_max_matrice_/4 has been adopted for checking the delamination, i.e., material fracture, a typical failure of laminate composites. The last criterion has also been adopted for checking the bonding. All stress analysis has been satisfied, as shown in Figure 6d–g: the achieved equivalent stresses are lower than 521 MPa, tensile strength of the adopted carbon fiber, and the achieved frictional stresses are lower than 85 MPa, debonding composites stress.

To prototype the foot, the skin-core structures are made of a 5 mm Easycell foam core between two carbon fiber fabric skins, pre-impregnated with epoxy resin, consisting of 3 layers of 3k twill XC110 oriented at 0°, +30°, and +60°, with respect to the longitudinal axis of the foot. In the C and F zones of Figure 6b, the 0° layer is missing in order to increase compliance. The two structures are glued using the same structural matrix used for the realization of the skins, and the inserts for joining, made in aluminum alloy ALU 6082-T6, to the other structural elements are also glued.

#### 2.2.2. Actuator and Transmission System Design and Assembling

The detailed design of the actuator/transmission system was performed prior to the construction of the connecting parts and the assembly of this prototype subsystem. The actuator needs to be controlled in velocity, with a good frequency response at 100 Hz, and should be able to maintain defined positions. The transmission should convert the rotational in a linear motion and it should be possible to have a compact transmission and engine assembly. In order to define the technical data of the actuator/transmissions system, the dynamic model has been used to calculate the values of speed, acceleration, force, and power required for the system in a gait cycle. In Table 4, the data obtained as output of the model are shown as maximum values.

**Figure 7:**
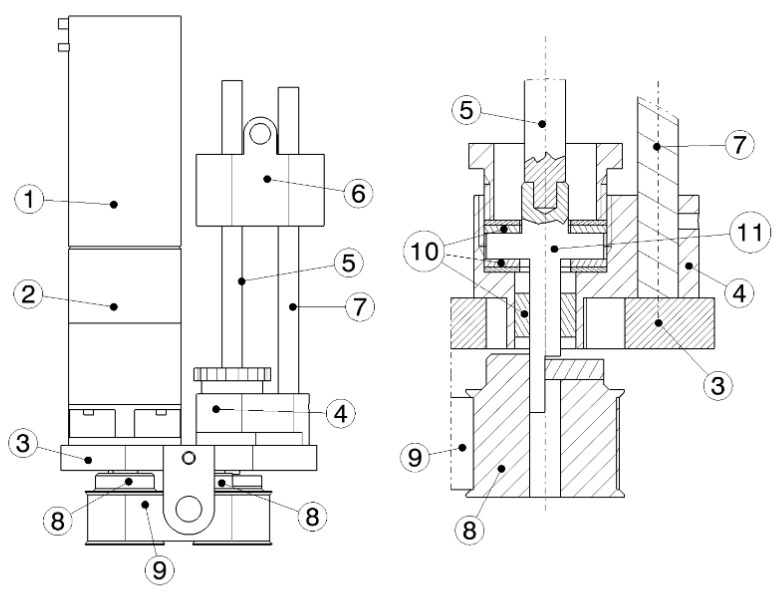
Details of the actuator/transmissions system: (1) rotary motor; (2) gearbox (1st transmission); (3) base plate; (4) screw frame; (5) roller screw (3rd transmission); (6) moving slider; (7) guide bar; (8) transmission pulleys (2nd transmission); (9) belt; (10) bearings; (11) axial loads frame.

The result of the design of the commercial components of the actuator/transmissions system is a Faulhaber brushless electric servomotor mod. 4490BS with 283 Watt at 24 Vdc of nominal voltage, very compact and light (diameter of 44 mm, length of 90 mm and mass of 742 g). This motor can rotate in both directions of rotation with speed up to 16,000 rpm and stops in position if the motor is not powered. The speed reducer with differential gear, Faulhaber mod. S44/1, has a transmission ratio equal to 1:44. The output shaft of the gearbox is then connected to a second transmission: a synchronous belt transmission between parallel axes has a transmission ratio of 1:1, which transports the output power on an axis parallel to that of the motor. The driven pulley is then connected to the shaft of a screw, with an axis parallel to that of the motor, which forms the third and last transmission: a screw-nut transmission converts the rotary motion in a translational motion. In Figure 7, some details of the actuator/transmissions system design with the main components present in the system are shown.

All the connecting components are made of ALU 6082-T6. To prototype the screw-nut transmission, a Misumi ball screw mod. C-BSSC Φ8 p4, whose maximum allowable load is a quarter of the roller screw, was the best choice in design, but it was unfortunately too expensive for the research budget. This reduction in performance has been considered during the experimental activity. By a RS232 serial interface, the actuator is connected and controlled by the Faulhaber MC 5010 S RS driver.

#### 2.2.3. Shock-Absorber, Rocker Arm Design and Prototyping

**Figure 8:**
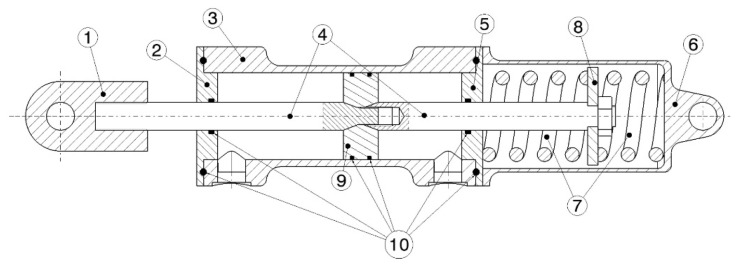
Details of the shock-absorber: (1) eyelet on the moving rod; (2) upper head; (3) body of the shock-absorber; (4) rods; (5) lower head; (6) compartment of the spring; (7) springs; (8) plate for interaction rod-springs; (9) piston; (10) seals.

The shock-absorber consists of a traction/compression elastic system and a viscous damper. It will allow the storage and the release of the elastic energy and should allow sagging of the foot in the presence of a dynamic load generated by an impact in the calcaneal area. It consists of a double-rod (one internal and the other one external) cushioning system (Figure 8), with an integrated pair of opposed springs. This solution allows for the assembly of the elastic element in parallel with the damper, to guarantee both traction and compression functioning and to keep constant the sum of the volumes of the two chambers of the damper, so that further storage chambers of fluid are not required. Furthermore, by means of an external system for the recirculation of the fluid, no orifices in the piston are necessary, and the damping effect can be adjusted by a servo drive that could adjust the level of lamination of the fluid between the two chambers and the stroke can be blocked. For the choice of materials, a 50-bar pressure value of the fluid has been assumed. Except for the rods, to be made of C45 SAE 1045 steel, all components are made of ALU 6082-T6. By means of an iterative procedure carried out with the stress analysis Ansys code, a diameter equal to 8 mm, bore equal to 25 mm, and lower thickness of the body of the shock-absorber equal to 1 mm have been defined. The total mass of the shock-absorber has been calculated to be approximately 600 g and the stroke was set to 60 mm.

The prototyping of the shock-absorber followed the detailed design. For reasons of simplification in the construction of this first prototype, having verified in quasi-static conditions that the damping effect is not necessary, the prototype of the shock-absorber was made only with the elastic elements. The latter integrates two drilled plates to be constrained to one end of the shock-absorber and to the moving rod of it in order to allow the same compression-tensile behavior. The lateral structural rods and the moving one are made of grinded carbon fiber tubes; the moving rod and one of the two eyelets are glued by the same structural adhesive adopted for the foot.

The rocker arm design was very simple. Rocker arm is made of two “boomerang”-shaped elements, joined by metal pins. Three ball bearings are housed in each element. The resulting shape and the thickness of such elements have been achieved by a topological optimization procedure carried out with the Ansys code. In prototyping the rocker arm, the Nylon PA12, instead of ALU 6082-T6, was used for two reasons. The first is a small reduction in the actuator compression/traction load compared to the design data, while the second is to use it as a mechanical fuse. In this way, if an extra load occurs during the experimental test, the rocker arm breaks, avoiding damage to the more expensive components of the prototype.

**Figure 9:**
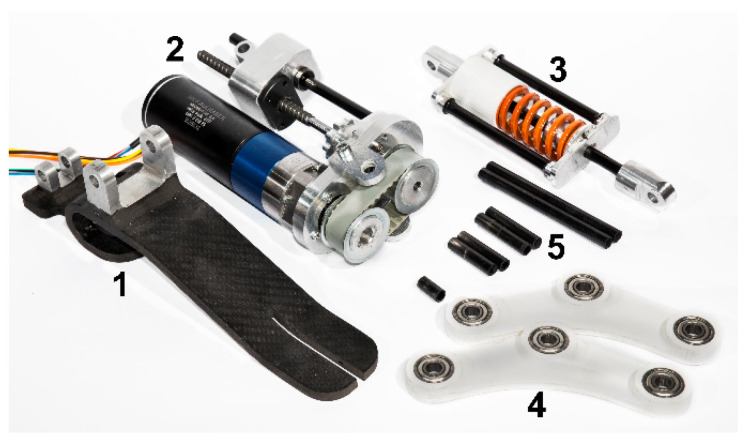
Components of the ankle-foot prosthesis prototype: 1. foot; 2. linear actuator and transmissions; 3. shock-absorber; 4. rocker arm; 5. structural pins.

The frame of the prosthesis has been made by two commercial 30 mm × 30 mm aluminum profiles connected to the two sides of the prototype. Two holes, carried out on each profile, allow the passage of the structural pins in correspondence with the ankle joint and the middle hinge of the rocker arm. Finally, the overall mass of the prototype of the ankle-foot prosthesis is equal to 2.23 kg, and Figure 9 shows all the prototyped components of the ankle-foot prosthesis.

## 3. Experimental Test Design and Results

The experimental activity on the prototype has been carried out in two steps with the goal of a functional validation of the design of the ankle-foot prosthesis: the first one, to test each component, the second one, for a preliminary check of the functionality of the whole prototype. For the first step, a vice of a hydraulic press has been adopted to fix the required component between its jaws, and a hydraulic cylinder has been adopted to apply the required vertical load. For the second step, an experimental set-up has been used to obtain the measurements related to the functional behavior of the ankle-foot prosthesis prototype.

### 3.1. Experimental Tests on the Components

Different types of tests have been designed and implemented for the components of the prosthesis. In particular, for the foot, the tests performed are shown in Table 5; for the other components, in Table 6.

### 3.2. Experimental Tests on the Complete Prototype

**Figure 10:**
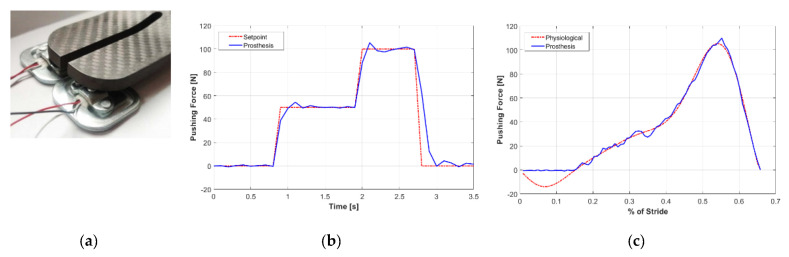
(**a**) Detail of the load cells under the distal parts of the foot of the prosthesis; (**b**) Pushing force in a step response test with the proportional, integrative and derivative (PID) control system; (**c**) Pushing force phisiological vs. prosthesis with the PID control system in the pre-swing phase.

Different types of tests have been designed and implemented for the ankle-foot prosthesis prototype. At the beginning, the measurement of the variation of the ankle angle was carried out, starting from the γ = 90° position of the prototype and the foot not in contact with the plane. The measurement of the range of variation of the ankle angle is equal to −15.4°–+12.2°. The other tests carried out on the prosthesis required the preparation of a laboratory set-up based on two compression load cells Worldchip 925119, fixed to the base plane, for measuring the pressure of left and right distal area of the foot. The signals were acquired by a DAQ board National Instruments USB-6001 and a software code in a LabView environment. For these tests, the tibia, positioned vertically, was made integral with a fixed frame and the foot was positioned horizontally, γ = 90°, with the distal area above the load cells (Figure 10a).

The first test was carried out to validate the ability of the prosthesis to apply and maintain a defined thrust value by a force control system. A PID control (proportional, integrative and derivative) of the force applied by the distal area of the foot on the ground was therefore carried out by acting on the position of the slide controlled by the motor. The coefficients of the PID controller were determined with the autotuning procedure present in LabView and are the following: K_p_ = 0.20, K_i_ = 0.15, K_d_ = 0.08. Several force steps were given as a step signal and in Figure 10b a typical result obtained in the laboratory tests is shown.

The second test, always carried out with the tibia attached to a fixed frame, is a comparison between the pushing force during the pre-swing phase, limited to the interval in which the foot is kept approximately parallel to the ground, and the pushing force in the physiological step. To obtain this change in strength in the physiological step, the trend of the torque at the ankle shown in Figure 4a was used. This physiological trend of the torque at the ankle was post-processed, to take into account the pushing force’s arm on the ground with respect to the ankle joint; then, it was further elaborated to first obtain the law of displacement of the point of application of the spring that acts on the hinge of attachment to the foot and, subsequently, going up in the flow of power, the law of displacement to be assigned to the motor of the prosthesis, to obtain the ankle torque trend for physiological walking. At this point, this law of motion was assigned to the prototype motor and the change in the pushing force of the distal part of the prosthesis on the plane was measured with the load cells. Figure 10c shows the result obtained from this comparison; however, the values over 40% of the stride are not significant as the foot of the prosthesis has maintained its horizontal position, while in the physiological step the foot changes its inclination.

## 4. Discussion

The considerations that emerge from the tests carried out on the prosthesis are useful as a preliminary validation of this new ankle-foot prosthesis design.

The tests on the carbon fiber foot made it possible to validate the construction of the component and the ability to manufacture the component with the correct lamination of the layers and the workmanlike execution of the resistant aluminum inserts. Since for this realization we turned to an external company, the good results suggest continuing the collaboration for the realization of the second prototype of the prosthesis. The contained measurements of the foot deformation in different configurations confirm the validity of the foot design and allow us to confirm that it will not undergo significant changes in the design of the second prototype. As regards the electric motor-transmissions group, the preliminary results show that the design is well-conceived, even if the choices of the components did not allow us to reach the design values: the maximum speed of 15 mm/s, instead of 200 mm/s, the acceleration of 800 mm/s^2^, instead of 3.000 mm/s^2^, while the 2 h test with an axial force of 1.000 N gave a positive result with respect to a design axial force of 1.600 N. These choices were conditioned by the cost of the components and the consideration that the first prototype served to validate the concept and the functionality of the prosthesis and therefore it was possible to derogate from some specifications. The test of the stiffness of the motor-transmissions group also gave a positive result and demonstrated that the prototype is adequate for the needs of this step of the research.

Moving on to the tests carried out on the assembled prosthesis, it is observed that the range of motion of the ankle angle is very close to that found in the walking of a healthy person: 27.6° vs. 29°. The most interesting tests were those carried out to determine the pushing force of the foot of the prosthesis by a force control system. These tests have shown the ability to apply a pushing force up to 100 Newton, certainly lower than that required for a real prosthesis but sufficient for the preliminary validation at this step of the research. The pushing force PID control system responds well in the step response with a dynamics response not far from that required: the rise time in the 50 Newton ramp was approximately 0.2 s with an overshoot of about 3 Newton. The small overshoot is not significant as the shock-absorber was made without the damper. The last test shows an interesting result that represents to some extent a validation of the design methodology used. The law of movement calculated by the model, starting from the ankle torque in a physiological gait, and assigned to the prototype motor allows for a valid matching with the physiological one for the pushing force, although within the limits of the pre-swing sub-phase, where the foot is kept in a position fairly close to the horizontal one. The deviation between the two curves in this phase is less than 5%.

The above-mentioned results, directly connected to the performance of the prosthesis, allow for further considerations. First of all, the developed design methodology allowed the authors to overcome the first difficulty encountered by anyone who wants to develop an ankle prosthesis: “how to design a powered ankle-foot prosthesis”. For the calculation of the ankle angle and the necessary torque, the knowledge of the kinematics of the gait and the contact force exchanged between the foot and the ground is certainly fundamental. With regard to the kinematics, we proceeded with experimental tests aimed at measuring the angles between the components of the lower limb: the design methodology can likely be simplified by referring to data tables taken from the scientific literature. Regarding the calculation of the contact force, the design methodology, in this work, referred to the C-Motion software. One could either, as in the previous case, refer to data taken from the scientific literature or proceed through experimental tests, on healthy subjects, carried out with modern gait analysis systems equipped with sensorized pads [18,19,20], which are able to provide the contact force and its point of application with high precision during a physiological gait. Regardless of how the aforementioned data is entered, the methodology makes us quite confident that the results provided will be able to satisfy the specific requirements.

Regarding the activation mode and the energy storage/release system, it can be said that solutions similar to those in this work have already been adopted: the first is typically based on electric motors, the second is made by means of an elastic element. However, the ankle-foot prosthesis proposed in this work is based on the combination of the electric motor and the shock-absorber system to continuously control, during the entire step, the torque to be applied to the ankle and the angle of it. Other comparisons with existing prostheses cannot be made: the latter, equipped with tested control systems, are already at an advanced level and have already been tested in clinical tests. On the contrary, this prosthesis is still in the initial stage of its development.

Several questions are still open. Although the performance of the ankle-foot prosthesis seems satisfactory from a technical point of view, it has yet to be demonstrated whether the proposed prosthesis is really of benefit to an amputee subject and if it actually reduces the typical increase in the metabolic cost [21] of an amputee walking, when compared with that of a healthy subject. The first aspect certainly applies to our prosthesis, but it concerns all the motorized prostheses under development [10]. In addition, as a specification also considered necessary by other researchers [22,23,24], the possibility of adjusting the ankle angle in the swing phase avoids the occurrence of stumbling or the sliding of the same on the ground or accidental collisions with obstacles. However, the prosthesis is not yet able to recognize the phases of the stride. Furthermore, it has been designed for walking on a horizontal plane, but for its effective use it must be able to allow the gait on a slope or on steps.

To solve the still open questions and, therefore, to validate the total effectiveness of the proposed device, the prototype must be built on the basis of the mechatronic design; therefore, the control system must be developed in order to detect the subject’s intention, to adapt the prosthesis to the conditions of the ground, and to recognize the phase of the stride. The control system described in the present work is aimed to check the capability of the prosthesis to develop the desired force in static conditions, regardless of the phases of the gait. Based on this and other considerations, the work steps to be done have already been identified, as described below: to develop a control system to be implemented first of all using the software in the loop method integrated with the developed inverse dynamic model; then, to create the physical controller to be implemented using the hardware in the loop method; finally, to integrate the controller into the real prosthesis to start an experimental pre-clinical activity with healthy subjects for the functional and dynamic characterization of the entire device and for its improvement. The last step of the research activity will be the validation of the prosthesis in a clinical campaign of tests with amputee subjects.

## 5. Conclusions

The biomechanical design and the construction of a first prototype of an innovative powered ankle-foot prosthesis with an energy storage inside is presented. It consists of a five link device integrated with two keys elements: a shock-absorber and a linear actuator-transmissions group. During the stance phase, the simultaneous operation of the shock-absorber and of the actuator allow for the recovery and transfer of energy for the push-off step; during the swing phase, the ankle angle is adjusted by the action of the actuator only. The elementary force control applied by the distal area of the foot is implemented on the prototype. The result of the preliminary tests in the laboratory on the prototype alone showed encouraging behavior and suggests continued research. Future developments require the prototype to be upgraded so as to comply with the design of the prosthesis in all its components. Furthermore, the control system must be developed in order to detect the subject’s intention, to adapt the prosthesis to the conditions of the ground, and to recognize the phase of the stride.

## 6. Patents

The ankle-foot prosthesis design was patented by the authors [25].

## Figures and Tables

**Table 1:** Demographic data of healthy subjects recruited for the gait analysis.

Id	Sex	Age	Mass (kg)	Height (m)
1	M	59	94	1.83
2	M	55	72	1.88
3	M	30	71	1.69
4	M	48	80	1.82
5	M	46	78	1.80
6	M	33	80	1.75
7	M	38	76	1.80
8	F	40	63	1.78
9	M	50	96	1.85
10	M	43	80	1.78
		44.2 ± 9.26	79.0 ± 9.98	1.80 ± 0.05

**Table 2:** Impact forces transmitted to the foot of the prosthesis in a gait cycle.

Area	Force (N)
heel	2.500
tarsal	2.500
distal	1.500

**Table 3:** Maximum force transmitted to the hinges of the prosthesis in a gait cycle.

Prosthesis Component	Force (N)
Foot (direction of the connecting hinges)	1.900
Shock-absorber (direction of the connecting hinges)	1.900
Actuator (direction of the connecting hinges)	2.000
Rocker arm (approximately vertical direction)	2.000

**Table 4:** Data for selecting the electrical motor and the transmission.

Kinematic and Dynamic Data	Values
Power	≥250 W
Assial force (both direction)	≥1600 N
Linear velocity	≥0.2 m/s
Acceleration	≥3 m/s^2^

**Table 5:** Tests for the foot prototype made of carbon fiber.

Tests	Results
* Quasi-static debonding test of the inserts* At foot fixed, a tensile ramp load, from 0 to 2.500 N (in compliance with the MFCL), has been applied in a time equal to 2 s and remained at the maximum value for 10 s. The load has been alternately applied on both inserts, on the calcaneal and tarsal area, 5 times for each one.	No debonding has been recorded.
* Dynamic debonding test of the tarsal insert* At insert fixed, a compression sawtooth load, from 0 to 1.500 N, has been alternately applied in a time equal to 2 s, 450 times, to the distal and calcaneal areas.	No debonding has been recorded.
* Measurement of the maximum static deflection of the tarsal area* At foot placed on a rigid surface, a compression static load of 2.500 N has been applied on the tarsal area.	A maximum deflection equal to 3.8 mm has been measured.
* Measurement of the maximum static deflection of the distal area* At foot fixed, a tensile ramp load, from 0 to 1.500 N (equal to the equivalent impact force in the distal area), has been applied on the overall distal area in a time equal to 2 s and remained at the maximum value for 10 s.	A maximum deflection equal to 7.4 mm has been measured.
* Measurement of the maximum differential deflection of the distal area* At tarsal area fixed, a compression 450 N static load has alternately applied, 10 times, on each distal area.	A maximum differential deflection equal to 1.2 mm has been measured.

**Table 6:** Tests for the actuator/transmissions system and for the shock-absorber.

Tests	Results
ACTUATOR/TRANSMISSIONS GROUP	
* Functional test with no load* Upward and downward cycles (overall stroke 50 mm) of the moving slider at the maximum speed, 15 mm/s, and acceleration, 800 mm/s^2^, continuously for 10 h.	No stop or overheating has been recorded.
* Functional test with load * In the same conditions of the previous test, an axial 1.000 N tensile load (2 masses of 50 kg) has been applied to the mobile slider for 2 h.	No stop or overheating has been recorded.
* Stiffness test of the screw and the guide bar* In the plane containing the axes of symmetry of the screw and the linear rail, a static compression 200 N load has been applied perpendicularly to the moving slider, moving at a constant speed equal to 4 mm/s.	No jamming has been recorded along the stroke of the moving slider.
* Test of the irreversibility of motion* At not powered motor and starting from the position corresponding to the center of the stroke of the moving slider, a compression and a tensile 600 N load has been alternately applied to the moving slider.	No motion of the slider has been recorded.
SHOCK-ABSORBER	
* Test to check for any jamming of the rod of the shock-absorber* At shock-absorber placed in vertical position with a fixed fork, a compression 600 N load has been applied to the other fork.	No jamming has been recorded along the stroke of the rod.
